# Large-Area Nanocrystalline Caesium Lead Chloride Thin Films: A Focus on the Exciton Recombination Dynamics

**DOI:** 10.3390/nano11020434

**Published:** 2021-02-09

**Authors:** Naomi Falsini, Nicola Calisi, Giammarco Roini, Andrea Ristori, Francesco Biccari, Paolo Scardi, Chiara Barri, Monica Bollani, Stefano Caporali, Anna Vinattieri

**Affiliations:** 1 Department of Physics and Astronomy, University of Florence, Via G. Sansone 1, I-50019 Sesto Fiorentino, Italy; francesco.biccari@unifi.it (F.B.); anna.vinattieri@unifi.it (A.V.); 2 European Laboratory for Non-linear Spectroscopy (LENS), University of Florence, Via N. Carrara 1, I-50019 Sesto Fiorentino, Italy; andrea.ristori@unifi.it; 3 Department of Industrial Engineering (DIEF), University of Florence, Via S. Marta 3, I-50139 Florence, Italy; nicola.calisi@unifi.it (N.C.); stefano.caporali@unifi.it (S.C.); 4 INSTM-Interuniversity National Consortium for Material Science and Technology, Via Giusti 9, I-50121 Florence, Italy; 5 Department of Information Engineering, University of Brescia, Via Branze, 38, I-25123 Brescia, Italy; g.roini@unibs.it; 6 Department of Civil, Environmental and Mechanical Engineering, University of Trento, Via Mesiano 77, I-38123 Trento, Italy; Paolo.Scardi@unitn.it; 7 Institute of Photonic and Nanotechnology (IFN)-CNR, LNESS Laboratory, Via Anzani 42, I-20133 Como, Italy; chiara.barri@polimi.it (C.B.); monica.bollani@ifn.cnr.it (M.B.); 8 Department of Physics, Polytechnic University of Milan, P.zza Leonardo 32, I-20133 Milano, Italy; 9 National Institute for Nuclear Physics (INFN-Firenze), Via G. Sansone 1, I-50019 Sesto Fiorentino, Italy

**Keywords:** inorganic halide perovskites, CsPbCl_3_, thin films, sputtering, high resolution photoluminescence

## Abstract

Caesium lead halide perovskites were recently demonstrated to be a relevant class of semiconductors for photonics and optoelectronics. Unlike CsPbBr_3_ and CsPbI_3_, the realization of high-quality thin films of CsPbCl_3_, particularly interesting for highly efficient white LEDs when coupled to converting phosphors, is still a very demanding task. In this work we report the first successful deposition of nanocrystalline CsPbCl_3_ thin films (70–150 nm) by radio frequency magnetron sputtering on large-area substrates. We present a detailed investigation of the optical properties by high resolution photoluminescence (PL) spectroscopy, resolved in time and space in the range 10–300 K, providing quantitative information concerning carriers and excitons recombination dynamics. The PL is characterized by a limited inhomogeneous broadening (~15 meV at 10 K) and its origin is discussed from detailed analysis with investigations at the micro-scale. The samples, obtained without any post-growth treatment, show a homogeneous PL emission in spectrum and intensity on large sample areas (several cm^2^). Temperature dependent and time-resolved PL spectra elucidate the role of carrier trapping in determining the PL quenching up to room temperature. Our results open the route for the realization of large-area inorganic halide perovskite films for photonic and optoelectronic devices.

## 1. Introduction

Research on new materials has recently focused on halide perovskites as highly promising semiconductors for advanced photonic and optoelectronic applications [1,2]. Indeed, caesium lead halide perovskites, described by the formula CsPbX_3_ (X = Cl, Br, I), are excellent active materials for coherent and incoherent light sources, sensors and innovative solar cells [3,4,5]. Their relevant electronic and optical properties (direct band gap, fine gap tunability by changing the halogen and alloying, high carrier mobility, defect tolerance, etc.) and, in particular, their higher thermal and chemical stability with respect to hybrid organic-inorganic perovskites [6,7,8], make them attractive for the development of highly performing light sources and for the integration in photonic structures. Among these fully inorganic perovskites, CsPbCl_3_ (band gap about 3.1 eV at room temperature [9]) is optimal for the development of light emitters in the blue spectral range, as a substitute of nitride-based materials, and it is the most suitable for the realization of white LEDs, coupled to a converting phosphor.

So far, solution-based techniques represent the most common route for the deposition of both organic-inorganic and fully inorganic perovskite films [7,10,11]. However, the main problems of these techniques are the limited scalability and/or the inhomogeneity of the deposited material, negatively affecting the large-scale production and external quantum efficiencies for light emission. Usually, to overcome the reduced material quality, the addition of chemicals is required in combination with post-deposition annealing. Moreover, to the best of our knowledge the deposition of CsPbCl_3_ films through dissolution of its precursor salts is not achievable. Different strategies for the synthesis of CsPbCl_3_ have been reported in literature: bulk crystals by the Bridgman method [12]; nanocrystals by high temperature solvent [9] and similar methods; thin films by evaporation [13,14,15,16,17]. It is worth mentioning that the possibility of realizing large-area compact films of nanometric thickness is relevant for the scalability of innovative devices. Moreover, the use of a technique like the sputtering discussed in this work, that allows for multilayers deposition (i.e., active material, electron/hole transport layers, metallic coatings, etc.) in a controlled atmosphere, over different substrates, opens the route for a wide set of applications in the field of photonics and optoelectronics, including the realization of metasurfaces.

In this work, following our recent paper on CsPbBr_3_ [18], we have obtained the first and successful deposition of nanocrystalline CsPbCl_3_ thin films by radio frequency (RF) magnetron sputtering. We present a detailed study of a set of samples with film thickness in the range 70–150 nm deposited on two different kinds of substrates. Beside morphological, structural and compositional analysis, we investigated the material optical properties by high resolution photoluminescence (PL) spectroscopy, resolved in time and space in a wide temperature range (10–300 K), providing quantitative information concerning the recombination dynamics of carriers and excitons. Our results demonstrate the macroscopic homogeneity of the CsPbCl_3_ thin films on large sample areas, which is hardly achievable, especially with a single step deposition, without the addition of specific chemicals and without post-growth treatments.

## 2. Materials and Methods

Several thin films of CsPbCl_3_ were deposited by RF magnetron sputtering onto two properly cleaned different substrates: soda lime glass (SLG) and amorphous quartz slides. The magnetron sputter used for sample preparation is a HEX system (Korvus Technology Ltd., Newington, UK) equipped with an RF source, a rotating sample holder to ensure the uniformity of the deposited film and a gravimetric microbalance to set the film thickness. The deposition was obtained in a non-reactive Argon atmosphere with a gas flow of 20 sccm (deposition rate 0.05 nm s^−1^). The sputtering of CsPbCl_3_ was obtained by a homemade target fabricated by mixing CsCl and PbCl_2_ precursor salts (Merck KGaA, Darmstadt, Germany) in equal molar ratio. The salts were dried in oven at 120 °C overnight and ground, by using a mixer mill (model MM400, Retsch, Haan, Germany), to obtain a uniform mixing. The mixture was then pressed at 11.5 MPa while the system was heated at 160 °C for the sintering of the powder. Further details on the sputtering procedure are reported in ref. [18].

Samples were characterized by scanning electron microscopy (SEM), atomic force microscopy (AFM), X-ray photoelectron spectroscopy (XPS) and X-ray diffraction (XRD) to assess morphology, crystalline structure and stoichiometry. Details concerning the used setups for these investigations are provided in the Supplementary Material.

XRD spectra were collected on a X’Pert diffractometer (Panalytical, Malvern, UK) equipped with CoKα X-ray source (40 kV, 40 mA), with a polycapillary optics in the primary beam (with 1 mm in equatorial direction and 10 mm height) and flat graphite crystal analyzer in the secondary beam, before the proportional counter. The grazing incidence measurements were made at a grazing incidence angle of 0.9° (actual incidence depends on the sample, as glass substrates are never perfectly flat). Beam divergence, as provided by the polycapillary lens, is 0.3°. Phase identification was based on the ICDD PDF-4+ database. All data, conventional θ/2θ and grazing incidence, were collected with a sampling step of 0.04° and counting time of 20 s. The XRD card matching the peak positions is #18-0366 of the ICDD PDF-4+ database (Tetragonal CsPbCl_3_, Space Group P4mm (99), unit cell parameters a = b = 5.584 Å, c = 5.623 Å), corresponding to a tetragonal crystal phase.

A Cary 300 spectrophotometer (Agilent, Santa Clara, CA, USA) equipped with a PELA-1050 integrating sphere (Labsphere, North Sutton, NH, USA) was used for the transmittance spectra of the samples at room temperature.

The PL of the deposited CsPbCl_3_ films was characterized by two different spectroscopy setups for detection at the macro and micro-scale. Most part of PL experiments was realized in a macro-PL configuration (laser spot diameter ≈ 100 µm) in a quasi-backscattering geometry, keeping the samples in a closed cycle cryostat and varying the temperature in the range 10–300 K. The excitation intensity was at maximum 10 W/cm^2^. A frequency-doubled mode-locked ps Ti:Sapphire laser, operating at 81.3 MHz repetition rate with 1.2 ps pulses, was used for time-integrated (TI) and time-resolved (TR) experiments: the excitation photon energy was varied in the range 3.3 to 3.45 eV. The fourth harmonic (266 nm) of a Q-switched Nd:YAG laser was used for excitation at 4.67 eV (repetition rate 20 KHz, 300 ps pulse duration). The PL signal was spectrally dispersed by a monochromator providing a spectral resolution of 1 meV and detected by a charge coupled device (CCD) detector (DU420-BU, Andor, Belfast, UK) for TI PL spectra or a synchroscan streak camera (C5680, Hamamatsu, Shizuoka, Japan) for TR measurements (time resolution ≈ 5 ps). Transmittance spectra in the temperature range 10–300 K were acquired simultaneously to PL by means of a white lamp.

For micro-PL experiments, the sample was kept at 10 K in a low-vibration ST-500 continuous He-flow cryostat (Janis, Lake Shore Cryotronics, Inc., Westerville, OH, USA) which in turn was mounted on an x-y translation stage (Physik Instrumente, Karlsruhe, Germany) for scanning the sample surface. The luminescence was collected by a home-made confocal microscope setup equipped with an infinity corrected 50× NUV objective (Mitutoyo, Neuss, Germany, 378-818-6, NA = 0.42). The luminescence was spectrally dispersed and detected using a SP2300i spectrograph (Acton, Teledyne Princeton Instruments, Krailing, Germany) equipped with two 1200 gr/mm gratings blazed at 350 nm and 750 nm, and an Acton Pixis 100F Si CCD (Teledyne Princeton Instruments, Krailing, Germany). The spatial resolution of the system is about 700 nm, while the spectral resolution is about 250 µeV. The excitation source was provided by a frequency-doubled mode-locked Ti:Sapphire tunable laser (Tsunami, Spectra Physics, Santa Clara, CA, USA, 700–900 nm spectral range, 200 fs pulse duration, 12.2 ns pulse period). The experiments were performed exciting the samples with photons of 3.40–3.45 eV and an excitation intensity at maximum of 100 W/cm^2^.

## 3. Results

### 3.1. Samples Characterization at Room Temperature

The samples investigated in this work are listed in Table 1: CsPbCl_3_ films of different thickness (70 and 150 nm) were realized on SLG and amorphous quartz substrates.

**Figure 1:**
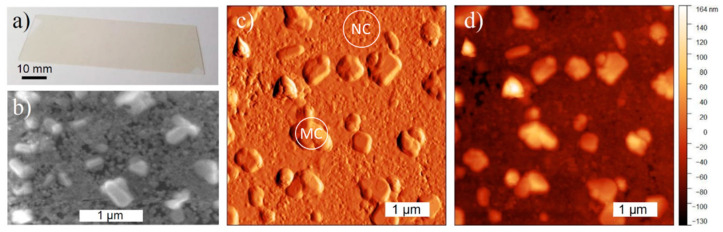
(**a**) Typical photograph of a 70 nm thick CsPbCl_3_ sample; (**b**) SEM micrograph of sample A. (**c**,**d**) AFM amplitude and topography maps of a 5 × 5 µm^2^ area of sample A. The circles with NC and MC indicate, as an example, regions with nanocrystals and sub-micrometer size larger crystal, respectively.

Highly uniformity and homogeneity over the substrate is always found at the macro-scale, as shown in the photograph of Figure 1a. The morphological characterization at the micro-scale is reported in Figure 1b–d, where a SEM micrograph and AFM analysis are respectively shown. They point out the presence of a fairly compact network of nanocrystals with average lateral size of 50 nm and average thickness of 40 nm just on the top of the substrate. Larger crystals (quite well isolated one from the other), up to several hundred nanometers size and with a height around 100 nm, are also found. Such values have been extracted by analyzing AFM profiles in different regions of the sample with an estimated uncertainty of ±10%. Hereafter, we identify with NC the network of nanocrystals and with MCs the sub-micrometer size larger crystals. Similar morphology is found from SEM investigation for samples differing in thickness or substrate.

**Figure 2:**
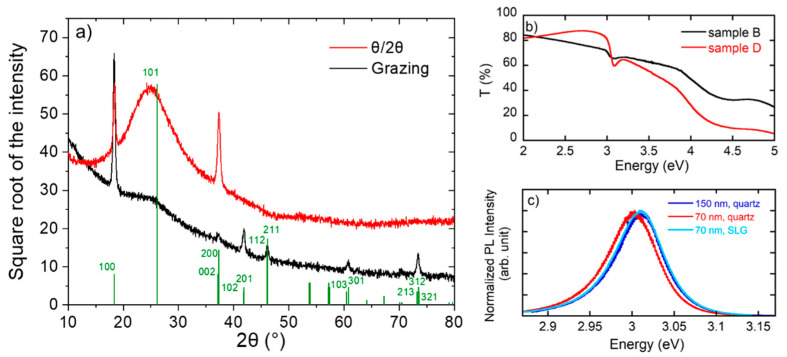
(**a**) XRD spectra of sample B in θ/2θ (red line) and grazing angle incidence (black line) configuration. The bars mark positions of the identified tetragonal phase. (**b**) Transmittance spectra of samples B (in black) and D (in red) at room temperature. (**c**) Normalized macro-PL spectra at room temperature comparing samples differing in thickness and substrate.

Phase identification was obtained via grazing angle incidence and θ/2θ XRD investigation. The XRD analysis (for details concerning the experimental apparatus see ref. [19]) clearly indicates the presence of the CsPbCl_3_ crystalline tetragonal phase (Figure 2a), as expected at room temperature [20,21], with a significant h00 fiber orientation which is commonly observed in our samples. The XRD results are compatible with the picture of a thin film made of two distinct perovskite fractions. In θ/2θ only (100) and (200) reflections are detected, while in grazing angle incidence other peaks are reported (Figure 2a). The broad peak at 26 degrees comes from the amorphous substrate. The θ/2θ diffraction pattern shows the signal from crystalline grains with (hkl) atomic planes parallel to the surface: besides the broad signal from the large fraction of the X-ray beam going through the thin film and being diffracted by the amorphous substrate underneath, the two strong h00 lines in the θ/2θ pattern are given by large and presumably (fibre) textured grains. In the grazing incidence condition, instead, the large crystals contribute much less, except for the 100 line. AFM and SEM images (Figure 1) show that the large grains, better crystallized to the point that in some sample regions they even assume a geometrical shape typical of single crystals, have a large spread of orientations, thus explaining the presence of a still intense 100 peak even in grazing condition. The remaining part of reflections observed in grazing incidence, however, are sensibly broader and belong to planes with different orientations, likely originating from the (nano)polycrystalline fraction, which appears randomly oriented or just weakly textured. Even though, given the instrumental conditions, it is not possible to precisely determine the size of the domains, the observed broadening is compatible with perovskite domain sizes of the order of several tens of nanometers in agreement with the AFM and SEM images.

XPS spectra (Figure S1) were used to estimate the stoichiometry of the film surface. The binding energy values of caesium, lead and chlorine are in accordance with the literature data [22]. Literature sensitivity factors were applied and the atomic ratio of the elements are reported in Table 2 for different substrate and same thickness. They show a lead excess and a chlorine lack respect to the expected values [23]. It is worth mentioning that the discrepancy between the measured stoichiometry and the expected one can be ascribed to the peculiar nature of the XPS investigation that provides information only on a few nanometers below the surface. Presence of oxygen and carbon can be evidenced by XPS spectra (Figure S1). While adventitious carbon contamination, appearing as soon as samples are exposed to atmosphere, can be claimed as carbon source, oxygen is more likely related to the presence of metallic oxides, as demonstrated by the presence of a peak at 529 eV (Figure S1). Moreover, we observe that the intensity of the oxygen peak is modestly reduced by argon sputtering which is consistent with the presence of compounds formed during film growth rather than as a result of post deposition atmosphere exposure. Unambiguous identification of these minor compounds is out of the scope of the present work, however, lead oxides like PbO or PbO_2_, are the most reasonable candidates. Similar results are found for all the investigated samples.

Transmittance spectra at room temperature are shown in Figure 2b for B and D samples. The transitions at 4.4 eV and 3 eV are evident in absorption, as expected and reported in previous works for caesium lead chloride films and single crystals [14,24,25]. In particular, the narrow dip at 3 eV is ascribable to the fundamental excitonic resonance, characteristic of CsPbCl_3_ [14,24,25].

### 3.2. Photoluminescence Study

The main focus of our work concerns PL properties to validate the good optical quality of the material. Different PL experiments were performed changing the spatial resolution of the PL setup, from 1 µm to 100 µm. A comparison of the PL emission at room temperature is shown in Figure 2c for samples differing in thickness/substrate (PL spectra at low T are shown in Supplementary Information in Figure S2). The PL line shape does not show relevant changes; only a slight shift of the PL peak energy, likely related to the different strain between the perovskite film and the substrate. More significant is the PL intensity change from spot to spot spanning a sample area of ~10 cm^2^ (Figure S2). While most of the spectra change in intensity of the order of 30%, a few cold spots are found with a reduced PL intensity.

#### 3.2.1. Photoluminescence at Macro and Micro-Scale

**Figure 3:**
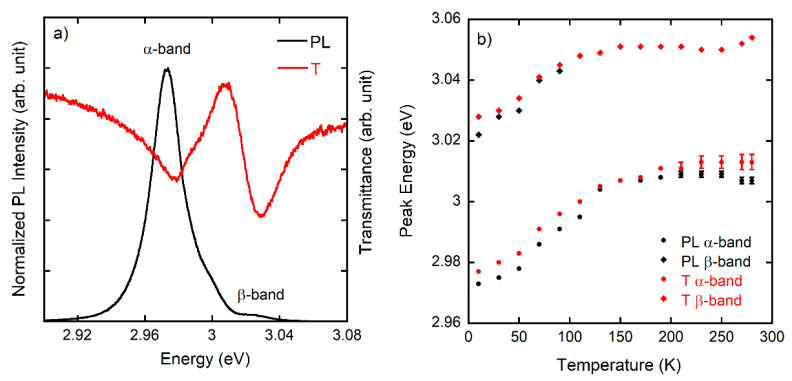
(**a**) Macro-PL (black curve) and transmittance (red curve) spectrum on sample A at 10 K after excitation at 4.67 eV. (**b**) Emission energy of the maximum of the PL for the α and β-band along with the corresponding minima of the transmittance spectra as a function of temperature for sample A.

To gain insights in the PL excitonic features low temperature spectra have been measured at micro- and macro-scale. A typical macro-PL spectrum of a CsPbCl_3_ sample on SLG substrate at 10 K is displayed in Figure 3a and compared with the high-resolution transmittance spectrum detected in the same sample spot. At 10 K the PL spectrum shows a dominant emission (α-band) peaked at 2.97 eV, in agreement with literature data on CsPbCl_3_ bulk and nano-crystals and thin films [26,27,28], and a less intense higher energy band (β-band) peaked at ≈3.02 eV which has also been reported in previous works [28,29]. At 10 K both bands, with a full width half maximum of about 15 meV comparable to single crystals [26], correspond to two resonances in the transmittance spectrum (Figure 3a) with a similar Stokes shift ≤5 meV, indicating an overall high quality of the emission that arises from two excitonic recombinations.

Figure 3b displays the temperature dependence of the PL peak energy of both bands and transmission resonances, confirming that excitonic features are observed up to room temperature. A decrease of the Stokes shift between PL and transmission is observed from 10 to 150 K. The macro-PL spectra as a function of temperature (*T*) (Figure S3) show, as expected, a blue shift of the spectrum and a quenching of the emission; moreover, in the α-band a structure appears at higher energy ascribable to a free exciton (FE) recombination around 100 K that becomes dominant as the temperature increases.

Power dependent PL spectra at 10 K reveal a linear dependence of the emission as expected for geminate (excitonic) recombination of carriers or when the radiative recombination prevails over the non-radiative one.

**Figure 4:**
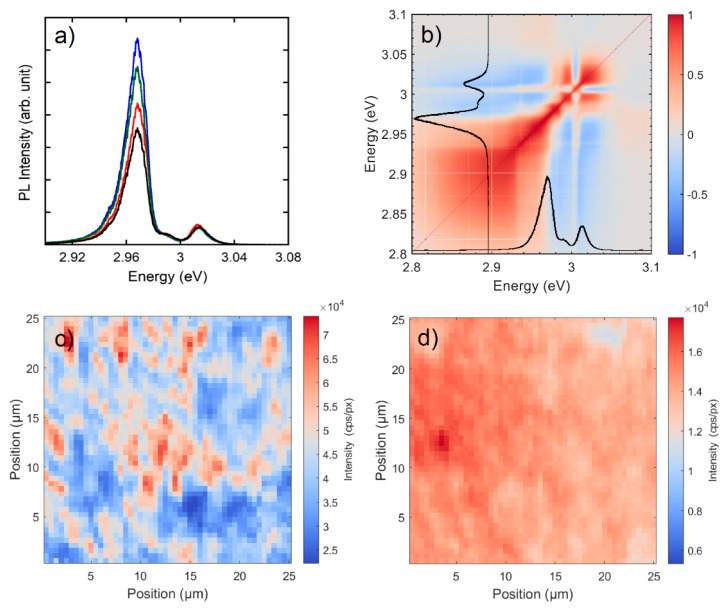
(**a**) Micro-PL spectra at 10 K for sample A acquired in different sample spots spanning a 25 × 25 µm^2^ area. (**b**) Pearson correlation map in the same sample area. The black lines correspond to the spatially integrated PL spectra in the same sample area. (**c**,**d**) PL intensity for P_α_ and P_β_, respectively, in the same sample area.

At low temperature macro-PL spectra show negligible sample inhomogeneity in terms of PL intensity of each band and peak energy shift. To deeply investigate the sample inhomogeneity so as to provide information on the origin of the inhomogeneous broadening (IB) we performed micro-PL experiments with a spatial resolution ≤ 1 µm. Typical results are shown in Figure 4a where we compare spectra in different points. Micro-PL spectra highlight a finer structure of the α-band with contributions in the low energy side and a shoulder in the high energy side, reported in Figure 4a. According to literature [27], the shoulder on the low energy side of α-band could come from a free exciton phonon replica or, more likely, to a bound exciton in a deeper defect. The shoulder present in the high energy side of the α-band is, instead, related to the β-band (see Pearson correlation map of Figure 4b). Hereafter the peaks of the two bands are indicated as P_α_ and P_β_. It clearly appears that micro-PL spectra have IB similar to the one of the macro-PL spectrum of Figure 3a; differences are observed in the relative emission intensity of α and β-band (Figure 4a). It has to be noted that no normalization has been applied to the spectra of Figure 4a so that the emission of β-band is constant in the investigated sample region. Figure 4b reports the Pearson correlation map (in the Pearson correlation map each pixel, identified by two energy values, E_1_ and E_2_, is calculated correlating the PL intensity at E_1_ with the one at E_2_ over the entire ensemble of spectra (2500 in our case) in the micro-PL map) in a 25 × 25 µm^2^ sample area, showing that the emission of α and β bands are spectrally uncorrelated, therefore excluding the attribution of the β-band to an excited state of the α-band. A comparison among micro-PL maps, AFM and SEM images (Figure 1b–d) and results from XRD spectrum (Figure 2a) suggests the attribution of α-band to MCs and β-band to NC. In Figure 4c,d the PL intensity variations for the α and β-band contributions are reported, respectively. β-band is constant in intensity in sample macro-areas (hundreds of µm^2^), while intensity fluctuations at most of a factor 3 are observed for α-band. The PL peak energy variation in the sample is at maximum ± 3 meV for P_α_ (Figure S4) and less than 1 meV for P_β_. No correlation emerges between the emission peak energy and PL intensity, as from the comparison between the maps of Figure S4 and Figure 4c. The micro-PL shows also that, for all the investigated samples, the largest contribution to the IB of PL at the macro-scale does not come from disorder detectable at the micro-scale: in fact, the spatial fluctuation of the PL peak energy (average value 2.969 eV and standard deviation 1.8 meV) accounts for ≈ 10% of the overall IB, the remaining part being related to a sub-micrometer disorder attributable to the size inhomogeneity of the MCs. Moreover, we exclude that the major part of the broadening comes from a homogeneous contribution given the long dephasing time measured in inorganic lead halide perovskites [30].

#### 3.2.2. Time-Resolved Photoluminescence

**Figure 5:**
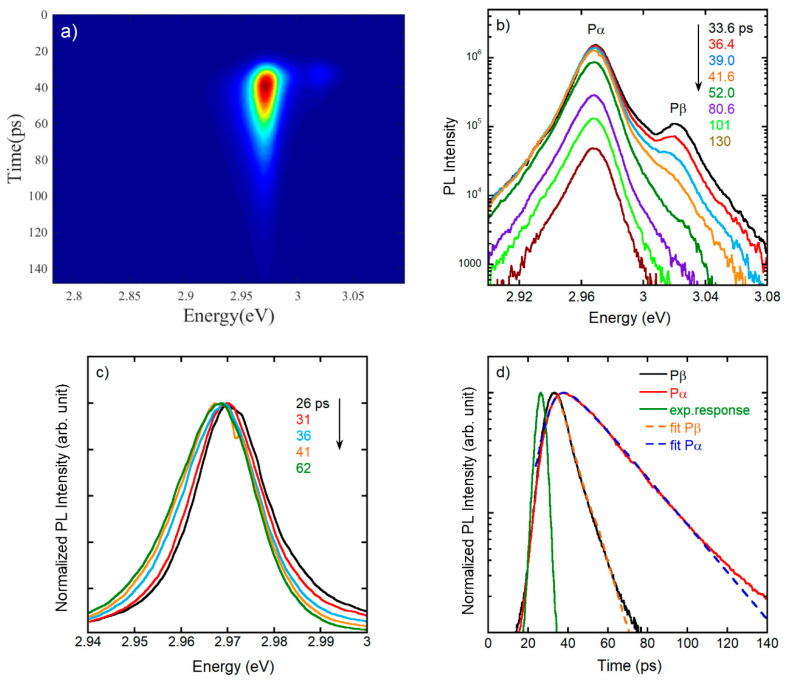
(**a**) Typical streak camera image of the time-resolved (TR) macro-PL at 14 K for sample A; (**b**) TR PL spectra extracted from a); (**c**) Normalized TR spectra showing the transfer of population from higher to lower energy states at increasing time delay; (**d**) PL decays (continuous line) extracted from (**a**) at peaks energy of P_α_ (in red) and P_β_ (in black) along with the experimental time response (in green). The fit functions (dashed lines) are shown superimposed to the experimental PL decays.

In order to clarify the nature of the recombination of α and β-band, we performed TR spectra at different temperatures (from 10 to 280 K) after picosecond excitation at 3.45 eV with an intensity of 10 W/cm^2^, corresponding to a photon flux of ~4 × 10^11^ photons/cm^2^. Results at 14 K are reported in Figure 5. A typical streak camera image is shown in Figure 5a, from which TR spectra and decays are extracted (Figure 5b–d). The TR spectra at different delay times are shown in Figure 5b on a log scale: all spectra, at each time delay, exhibit an exponential thermal tail whose effective time dependent temperature can be evaluated assuming the Boltzmann distribution for the carriers [31,32]. In this case the initial carrier temperature around 140 K progressively decreases down to 80 K (see Figure S5), maintaining a value higher than the lattice temperature for a long time, as already observed in CsPbBr_3_ [32]. After a fast rise the population thermalizes and, as a consequence, all the states around P_α_ (in the range 2.960–2.990 eV) decay with the same time constant. Instead, a strong difference of the PL lifetime is observed at P_α_ and P_β_ (Figure 5d), and the TR spectra of Figure 5b indicate a substantial independence of the two bands recombination with a lack of population transfer between β and α bands, confirming the absence of correlation of the two (Figure 4b).

The initial change in the spectral shape of α-band, due to thermalization, is shown in Figure 5c where we can easily notice that by increasing the time delay the population transfers from higher energy, corresponding to FE, to lower energy states, i.e., to localized or bound exciton states. After nearly 60 ps the PL decreases in intensity without further change in its shape, attesting the achievement of the thermalization. It is to remark that the TI spectrum at low *T* has a peak energy at P_α_, corresponding to a bound exciton recombination. Further evidence of such attribution comes from temperature-dependent spectra showing increased contribution of FE at higher temperature (Figure S3).

The PL time evolution at P_α_ and P_β_, at short times, and the corresponding fits are shown in Figure 5d, along with the experimental time response. In the fastest time scale, up to 100 ps, a single exponential decay with a time constant τ_1_ can substantially describe the PL time evolution. Each fitting curve is obtained as convolution between the experimental time response and the PL decay function, using an exponential rise and decay; the time constants for P_α_ are τ_rise_ 4 ps and τ_1_ 22 ps, while for P_β_ τ_rise_ is resolution limited and τ_1_ is 7 ps. TR PL data for all the investigated samples do not show remarkable changes in the time constant for α and β-band. It is also worth to remark that no change into the PL dynamics was observed by changing the excitation power over two orders of magnitude, between 1 and 100 W/cm^2^.

## 4. Data Analysis and Discussion

Our TR data prove that α and β emissions, which are present in all the investigated samples with similar features in terms of spectral line shape, relative intensity and time evolution, do not originate from a relaxation of higher energy (β-band) to lower energy states (α-band). This is an additional confirmation that they do not originate from the same spatial region in agreement with results of Figure 4b. Moreover, in this context, the significant faster decay of P_β_ respect to P_α_ is naturally explained by the major role played by surface state recombination in the NC as expected by the increased surface over volume states when the crystal size decreases [33].

In the comparison of our PL results with literature data on single crystal and amorphous films, it turns out that the main peak P_α_ (≈ 2.97 eV at 10 K) corresponds to a localized exciton recombination of CsPbCl_3_ [26,27].

More controversial is the assignment of the emission at ≈3.02 eV (β-band) already observed in single crystals, nanocrystals and amorphous films. This band is reported in previous works with different attributions; Ito et al. assign the β-band to the 2S excitonic transition [29], whereas Lohar et al. to a bound exciton [28]. Kondo et al., performing experiments at 77 K and high excitation density (>10 kW/cm^2^) observes a band at 2.97 eV with a superlinear behavior claimed as stimulated emission, while the FE emission is assigned to a band at ≈3.03 eV [34]. Our data exclude all the previous assignments of the β-band. Indeed, concerning the 2S recombination we have proven in TR spectra and in micro-PL maps that the β-band has no correlation in time and space with P_α_. Moreover, the power dependence of the PL intensity and excitation density used in our experiment exclude the presence of stimulated emission in the spectra. It has to be noted that β-band cannot be attributed to the precursor salts [35,36]. Moreover, the analysis of micro-PL maps (Figure 4) exclude any intermixing between α and β-band, corresponding respectively to the MCs and to the NC with a lack of “communication” between the two kind of emitters. We remark that such spectral features are always found in our samples, even though with different intensity, indicating that they are related to the growth conditions. To explain the different energies of the α and β-band several effects can be invoked: first of all, given the size of NC and MCs and the exciton Bohr radius of 2.5 nm [9], quantum confinement effects must be excluded provided that they are relevant only for NC with dimensions smaller than 10 nm. A possible origin could be related to the presence of a different strain between the substrate and NC and MCs, that show, however, an equal temperature dependent behavior (Figure 3b). We can envisage also two other possible origins of the different bands: a change in the stoichiometry of Cs, Pb, Cl [37] or different crystalline phase of CsPbCl_3_ in the MCs and NC, in analogy with reports on hybrid perovskites [38,39,40]. Two recent papers [41,42] highlighted structural changes crossing between NC and MCs and therefore it is very likely to suppose that the different energies of the α and β bands reflect such changes. Presently, we cannot experimentally distinguish between the previous hypotheses about the origin of the β-band: however, it must be pointed out that our data clearly show the formation of extended electronic states as certified by the excitonic resonances of Figure 3.

**Figure 6:**
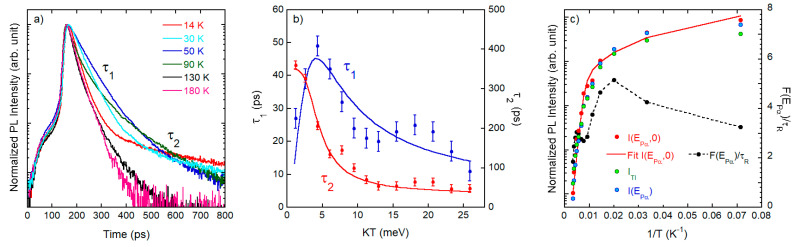
(**a**) PL time decay of P_α_ at different temperatures over an extended time range. (**b**) Temperature dependence of τ_1_ and τ_2_ along with fits (solid lines). All data refers to sample A. (**c**) Arrhenius plots extracted from the PL intensity vs. *T* along with the fit (solid line); *T* dependence of the PL time evolution factor *F(E_Pα_*)/*τ*_R_ in linear scale. The dashed line is a guide for the eyes.

A relevant aspect of our work concerns the temperature-dependent TR data and, in particular, the relative contribution of radiative and non-radiative recombination, providing quantitative estimate of the PL yield. In Figure 6a the PL time evolution at P_α_ for different temperatures is reported, over an extended range with respect to Figure 5d, showing a non-exponential behavior: for each temperature the decay can be nicely fitted with two exponentials with time constants *τ*_1_ and *τ*_2_*,* and the results are plotted in Figure 6b. The fast decay time *τ*_1_, after an initial rise at low temperature (*T*), decreases to smaller values, but the overall variation in the range 10–300 K is less than a factor 5; similar limited variation is observed for *τ*_2_; the small increase of both time constants observed for *T* > 150 K can be ascribed to the second order phase transition reported around this temperature for CsPbCl_3_ [43,44,45]. The fast initial decay time constant agrees with results of [46]. In Figure 6c we report different Arrhenius plots extracted from the PL intensity as a function of *T* that gives account of the PL quenching. In Figure 6c *I_TI_* indicates the PL intensity of the α-band integrated in time and over all the spectrum of the band itself, *I*(*E*_*P*__*α*_) is the TI PL intensity at P_α_ peak energy *E_Pα_*, and *I*(*E*_*Pα*_,0) is the maximum of intensity at *E_P_*_α_ as obtained from the TR spectra at different temperatures. The quantity *F*(*E*_*Pα*_)/τ_R_ will be defined in the following. Similar trends concerning the temperature dependence of the PL intensity are found for all the investigated samples. Despite the loss of PL intensity increasing the temperature (Figure 6c), the emission at room temperature is easily detectable. In order to correctly analyze the data, it is necessary to consider not only the variation with *T* of the PL intensity, but also the change in the PL time evolution. Indeed, under the assumption that the overall dynamics of the states involved in the radiative recombination is linear at a given temperature *T* (as proven by power-dependent measurements previously discussed), the time evolution of the PL intensity *I*(*E*,*t*), at a given energy *E*, is proportional to:

(1) $$I\left(E,t\right)\propto \frac{N\left(E\right)}{\tau_{R}}\int_{0}^{t}S\left(t^{\prime }\right)F\left(E,t-t^{\prime }\right)dt^{\prime }\;$$

where *S*(*t*) represents the time evolution of the exciting laser pulse, or more generally the response function of the experimental setup, *N*(*E*) the initial population of the excitonic states and *F*(*E*,*t*) the function describing the PL time evolution normalized to 1 at *t* = 0 (i.e., *F*(*E*,0) *=* 1). *τ*_R_ is the radiative time that, as a consequence of the increasing population of the excitonic states outside the light cone, scales as $T^{3/2}$ in bulk samples [47]. If *S*(*t*) is short enough with respect to the decay time, we can assume *S*(*t*) = *δ*(*t*) and then:

(2) $$I\left(E,t\right)\propto \frac{N\left(E\right)F\left(E,t\right)}{\tau_{R}}$$

Therefore, in a TI measurement we get:

(3) $$I\left(E\right)\propto \frac{N\left(E\right)F\left(E\right)}{\tau_{R}}$$

where *F*(*E*) is the integral over t, between 0 and infinite, of *F*(*E*,*t*).

In general both *N(E)* and *F*(*E*,*t*) depend on temperature (*T*): as a consequence, from the Arrhenius plot of TI PL data we cannot distinguish between a quenching originating from *N(E)*, i.e., from a decrement of the initial filling of the state, or from *F*(*E*,*t*), i.e., from a temperature evolution of radiative state dynamics.

In Figure 6c we report the Arrhenius plot of the PL intensity of the α-band integrated in time and over all the spectrum (indicated as *I_TI_*), of the TI PL intensity at P_α_ peak energy *E_Pα_* (i.e., *I*(*E*_*Pα*_) = *N*(*E*_*Pα*_) × *F*(*E*_*Pα*_)/*τ*_R_), and of the maximum of intensity at *E*_*Pα*_ as obtained from the TR spectra at different temperatures (i.e., *I*(*E*_*Pα*_,0) = *N*(*E_Pα_*)/*τ*_R_). On a linear scale we also plot the *T*-dependence of *F*(*E*_*Pα*_)/*τ*_R_, as obtained from the TR PL at *E_Pα_* at the different temperatures.

The strong resemblance in the T-dependence of *I_TI_* and *I*(*E*_*Pα*_) brings evidence that the thermal quenching is the same for all the states responsible for the α-band. Instead, the comparison between the Arrhenius plot of *I*(*E*_*Pα*_) and *I*(*E*_*Pα*_,0) gives account of the different contributions to the thermal quenching: provided that the variation of *F*(*E*_*Pα*_)/τ_R_ with *T* is quite small, it turns out that the main PL quenching comes from *N*(*E*_*Pα*_), i.e., from a decrease of the initial exciton population due to a fast (less than a few ps) free carriers capture in traps, before they reach the bottom of the band. In Figure 6c we show a fit of *I*(*E*_*Pα*_,0) obtained with the expression:

(4) $$I\left(E_{P\alpha ,0}\right)\;=\;\frac{N\left(E_{P\alpha }\right)}{\tau_{R}}\;=\;\frac{A}{{\left(kT\right)}^{3/2}\left(1+Be^{-\frac{E_{A}}{kT}}\right)}$$

where *E_A_* represents the effective activation energy of the capture center for the free carriers and the factor ${\left(kT\right)}^{3/2}$ accounts for the variation of τ_R_ with *T*. In Figure 6b we report τ_1_ and τ_2_ as obtained from the fits of the PL decay as a function of *T*, and the corresponding fits obtained with a standard two-level model describing the dynamics of an upper radiative state U interacting with a lower dark state L [48]. The fast time constant τ_1_ comes mainly from that of the upper state *U* and is given by the parallel of a non-radiative (τ_NR_) and a radiative (τ_R_) time constants. The non-radiative rate 1/τ_NR_ is assumed to vary with *T* as exp(−E_U_/kT) and the radiative rate 1/τ_R_ to scale as 1/(kT)^3/2^, according to data and theory for bulk systems [47]. The longer time constant τ_2_ is essentially the decay time of the lower state *L*, commonly attributed to a dark exciton [49], that thermally repopulates the upper one; the refilling rate from *L* to *U* is assumed to be thermally activated and then varying with *T* as exp(−E_L_/kT). From the fit a quite long refilling time at low *T* is obtained, i.e., ≈125 ps, and an activation energy E_L_ = 16 meV. For the activation energy of the non-radiative rate of the upper state we get from fit E_U_ = 7 meV, in agreement with the exciton localization energy (≈ 7 meV). In other words, by increasing *T*, excitons are promoted from localized to extended states and then are quickly captured by non-radiative recombining centers. For the radiative rate of the upper level from the fit we get τ_R_ = 40 ps at low temperature that, taking into account the proper scaling with *T*, corresponds to τ_R_ = 450 ps at room temperature, in agreement with literature [50,51]. Therefore, the strong decrease of the PL yield has to be ascribed, rather than to the exciton recombination, mainly to the reduced number of excitons that initially populate the radiative state: as a consequence, the presence of efficient traps for free carriers has to be invoked to explain the experimental findings as already found [52,53]. From this point of view, the observed presence of O and C in the XPS spectra (in particular O) could be responsible for traps. The role of oxygen in halide perovskites (both hybrid and inorganic) has been investigated by several authors. On the one hand in hybrid perovskites oxygen promotes the material decomposition [54], while in CsPbBr_3_ it can produce passivation of surface states, that increases the PL yield [55], or, depending on the material nanostructure, a detrimental effect [56,57]. As an alternative, or in addition to this, also a Pb rich and a Cl defective surface can provide efficient non-radiative recombination [49,51].

In our samples, having measured the PL yield at room temperature, from the Arrhenius plot, we estimate a PL yield at 10 K of ≈90% which agrees with what expected from the radiative and non-radiative rate at low *T* (Figure 6b).

## 5. Conclusions

We have demonstrated that fairly compact nanocrystalline thin films of CsPbCl_3_ can be realized by RF magnetron sputtering with high homogeneity in terms of optical properties with at most a variation of the PL energy position of the order of 0.1% over ~10 cm^2^. Samples prepared with this technique without any post-growth treatment and in absence of a polymeric coating show very good PL emission. To our knowledge, such homogeneity results are not achievable with standard deposition techniques without the addition of specific chemicals. In particular, the sample high optical quality is proven by the presence of limited IB (~15 meV). Detailed PL investigation provides quantitative information concerning the contribution of radiative and non-radiative recombination in CsPbCl_3_; the loss of PL yield comes from efficient capture of carriers in traps during the relaxation path. The presence of oxides could be responsible of such traps.

In conclusion, our results envisage the possibility of the use of RF magnetron sputtering for integration of fully inorganic perovskites in multilayer nanometric structures, such as photonic cavities, resonators and optical circuits.

## Figures and Tables

**Table 1:** Name, thickness and substrate type of investigated samples.

Sample	Average Thickness (nm)	Substrate
A	70	SLG
B	70	Quartz
C	150	SLG
D	150	Quartz

**Table 2:** XPS experimental atomic ratio and expected values for samples C and D.

Element	Glass (Sample C)	Quartz (Sample D)	Expected
Cs	20%	19%	20%
Pb	30%	30%	20%
Cl	50%	51%	60%

## Data Availability

The data presented in this study are available on request from the corresponding author.

## Supplementary Materials

Additional experimental details and data concerning XPS, SEM, AFM and PL are available online at https://www.mdpi.com/2079-4991/11/2/434/s1.

## Abbreviations

PL	Photoluminescence
SLG	Soda Lime Glass
RF	Radio Frequency
SEM	Scanning Electron Microscopy
AFM	Atomic Force Microscopy
XPS	X-Ray Photoelectron Spectroscopy
XRD	X-Ray Diffraction
TI	Time-integrated
TR	Time-resolved
NC	Network of nanocrystals
MCs	Sub-micrometer size larger crystals
FE	Free Exciton
IB	Inhomogeneous Broadening
