# Supplementary material for: Large-Area Nanocrystalline Caesium Lead Chloride Thin Films: A Focus on the Exciton Recombination Dynamics
Source: Nanomaterials (Basel). 2021 Feb 9;11(2):434. doi: 10.3390/nano11020434 (PMC7915012; doi:10.3390/nano11020434)
Supplement: Supplementary file 1 [file nanomaterials-11-00434-s001.pdf]

Supplementary Materials

Large-Area Nanocrystalline Caesium Lead Chloride Thin Films: A Focus on the Exciton Recombination Dynamics

Naomi Falsini [1,2,*], Nicola Calisi [3,4], Giammarco Roini [5], Andrea Ristori [2], Francesco Biccari [1,2], Paolo Scardi [6], Chiara Barri [7,8], Monica Bollani [7], Stefano Caporali [3,4] and Anna Vinattieri [1,2,9]

*Correspondence: naomi.falsini@unifi.it

Scanning electron microscopy (Philips XL30 SFEG SEM 30KV) and atomic force microscopy (Veeco Innova AFM, tapping mode scans with ultrasharp tips - radius of curvature of 2 nm) were performed to assess the morphology of the samples.

The chemical composition of the sample surface was determined using X-ray photoelectron spectroscopy (XPS) with an X-ray source (VSW Scientific Instrument Limited model TA10, Mg Kα radiation, 1253.6 eV) and a hemispherical analyzer (VSW Scientific Instrument Limited model HA100) with a 16 channels detector. To acquire the survey scan, the pass energy was fixed at 44 eV, for the high-definition spectra at 22 eV. The peaks were fitted using CasaXPS software and the background was subtracted using the Shirley's method [D. A. Shirley, Phys. Rev. B 1972, 5, 4709]. The binding energies values were calibrated using as internal reference the 1s transition of adventitious carbon fixed to 284.8 eV [P. Swift, Surf. Interface Anal. (1982) 4, 47].

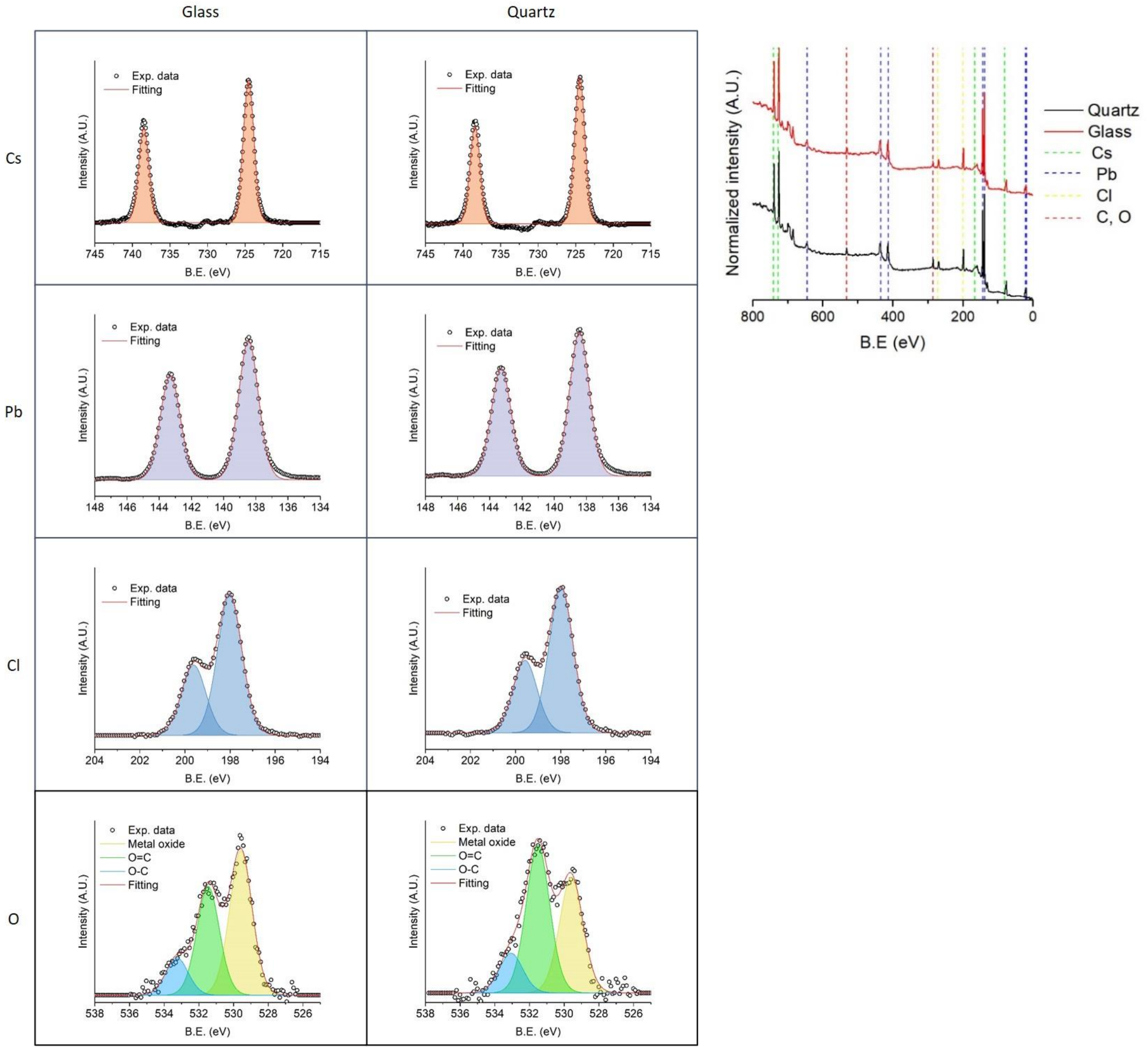

Figure S1. Left) XPS spectra of C and D samples, showing caesium (Cs), lead (Pb), chlorine (Cl) and oxygen (O) peaks; Right) Extended XPS spectra of the two samples. BE: Binding Energy.

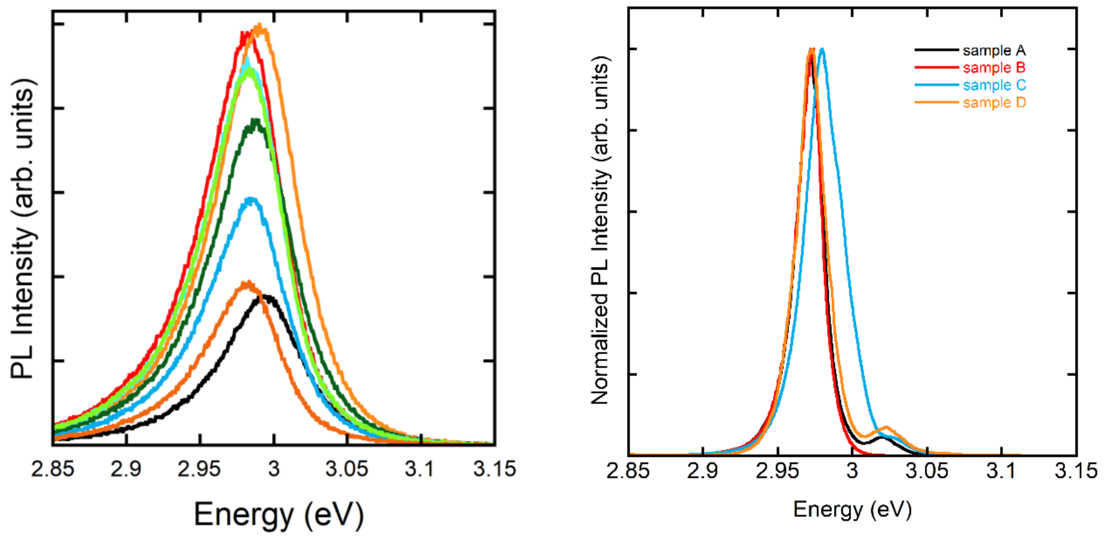

Figure S2. Left) Macro-PL spectra at room temperature in different spots of sample A, spanning an area of ~10 cm². Right) Macro-PL spectra of all samples at nominal 10 K. The shift of sample C respect to the other samples can be due to a different strain between the film and the substrate coming from a sample higher effective T (50 K).

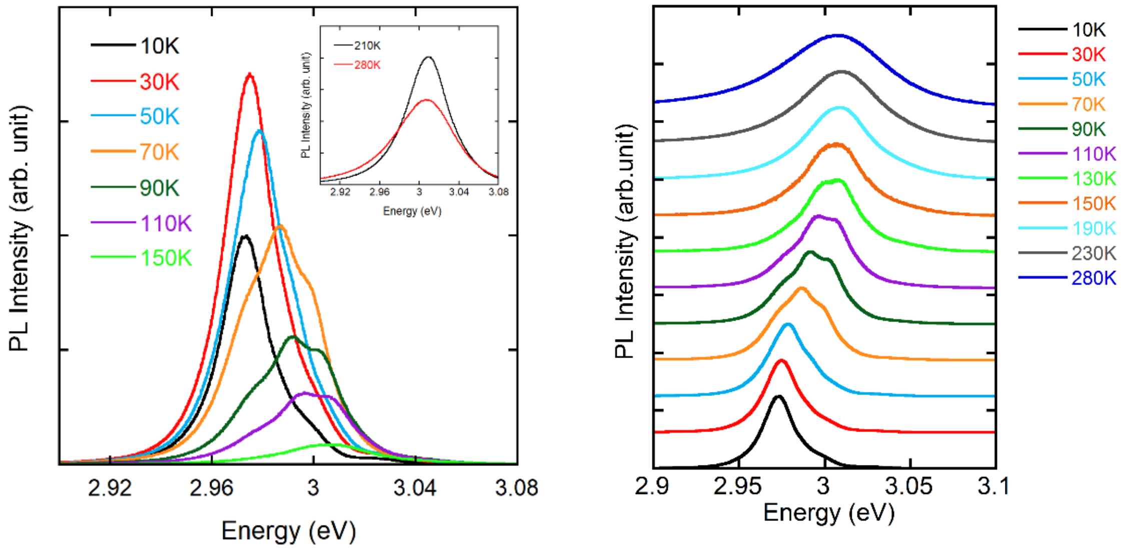

Figure S3. Left) Macro-PL spectra of sample A as a function of temperature showing the PL quenching. In the insert the high temperature spectra are shown. Right) Vertically shifted macro-PL spectra of sample A as a function of temperature showing the peak energy shift.

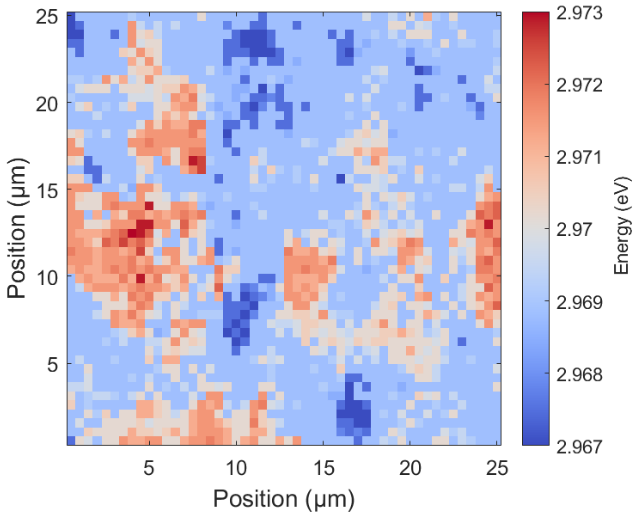

Figure S4. Micro-PL map reporting the variation of the P_{α} peak energy value in the same sample area of Figure 4 (main text).

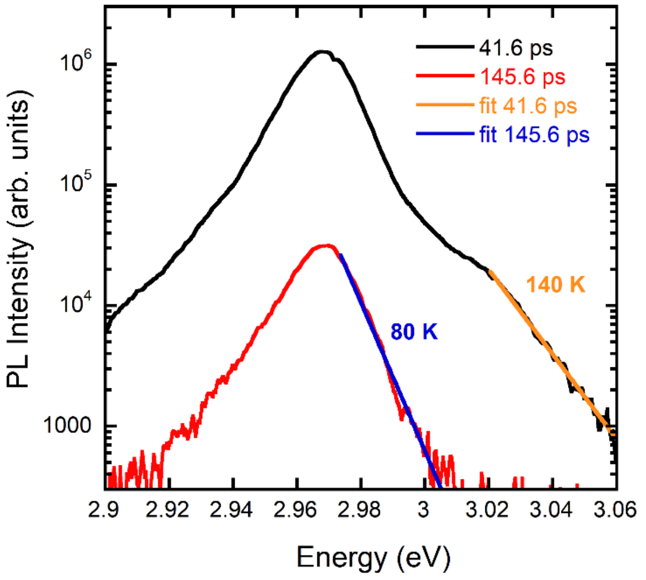

Figure S5. Time resolved spectra of sample A showing the exponential thermal tail at two different time delays.
